# Hemostatic Activity of Orally Administered Melanin from Cuttlefish Ink Sacs in a Rat Tail-Bleeding Model

**DOI:** 10.3390/molecules31203654

**Published:** 2026-10-13

**Authors:** Hao Han, Haobo Yuan, Yuanyuan Wang, Yuting Tan, Ching Yuan Hu, Qingbin Guo

**Affiliations:** 1 Shaanxi Provincial Bioresource Key Laboratory, College of Biological Science and Engineering, Shaanxi Province Black Organic Food Engineering Technology Research Center, Shaanxi University of Technology, Hanzhong 723000, China; yuanhaobo@snut.edu.cn (H.Y.); wangyuanyuan@snut.edu.cn (Y.W.); tanyuting@snut.edu.cn (Y.T.); 2 Department of Human Nutrition, Food and Animal Sciences, College of Tropical Agriculture and Human Resilience, University of Hawaii at Manoa, 1955 East-West Road, AgSci. 415J, Honolulu, HI 96822, USA; chinghu@hawaii.edu; 3 State Key Laboratory of Food Nutrition and Safety, Tianjin University of Science and Technology, Tianjin 300457, China

**Keywords:** cuttlefish ink sac by-products, hemostatic mechanism, metabolites in the small intestine, Mg^2+^ and Ca^2+^, UHPLC-Q-Orbitrap HRMS

## Abstract

Cuttlefish ink sac is an underutilized marine processing by-product with considerable potential for value-added conversion into functional ingredients. In this study, high-purity cuttlefish melanin (HCM) was isolated from cuttlefish ink sacs, and its hemostatic activity, safety, and intestinal metabolites were investigated to support its functional application. SEM-EDS analysis shows that HCM consisted of black spherical nanoparticles enriched in calcium and magnesium. Multi-technique characterization (UV–Vis, FTIR, XPS, and Raman) confirmed the material as melanin, with characteristic optical absorption, surface functional groups, and elemental composition. In a rat tail-bleeding model, HCM significantly reduced blood-loss volume and shortened bleeding time, indicating promising hemostatic activity in vivo. To explore the underlying mechanism, intestinal metabolites of HCM were characterized by UHPLC-Q-Orbitrap HRMS-based metabolomics. TXA and ACA were detected in the intestinal contents following HCM administration. Additionally, HCM released Ca^2+^ and Mg^2+^, which may contribute to regulating coagulation. Consistently, HCM significantly shortened prothrombin time and increased fibrinogen levels, while other coagulation parameters remained within the normal range. These results suggest that HCM may exert hemostatic effects through dual pathways involving fibrinolysis inhibition and coagulation promotion. Overall, this study provides mechanistic insight into the hemostatic activity of cuttlefish melanin and supports the high-value utilization of cuttlefish ink sac by-products as a source of marine-derived functional ingredients.

## 1. Introduction

The sustainable use of marine processing by-products has become a key issue in developing value-added foods and bioproducts, particularly given that global marine processing generates approximately 32 million tons of by-products annually, representing 50–70% of total catch weight [1,2,3,4]. During cuttlefish processing, ink sacs are often discarded or underutilized, despite being a rich source of melanin, a bioactive pigment with potential health benefits [5]. Recovering and using cuttlefish ink sac by-products may offer both economic and environmental advantages [6].

Postoperative blood loss is a common perioperative complication that may delay recovery, aggravate anemia, increase transfusion requirements, and ultimately worsen patient outcomes [7,8]. Perioperative anemia is a well-recognized risk factor for postoperative morbidity and mortality, and excessive bleeding remains a major challenge in surgical care [9,10,11]. Consequently, identifying safe and effective strategies for managing blood loss is of considerable interest. In addition to pharmacological interventions, naturally derived functional ingredients have received increasing attention for their potential safety, dietary compatibility, and value in nutritional regulation.

Hemostasis depends on the coordinated actions of coagulation, platelet activation, and fibrinolysis [12,13]. Tranexamic acid (TXA) reduces perioperative blood loss by inhibiting plasminogen activation and plasmin-mediated fibrin degradation [7,14,15,16,17]. However, there is continued interest in identifying safe, food-compatible hemostatic agents.

Melanins are natural macromolecular pigments found in various edible materials, including cuttlefish ink. Cuttlefish melanin is primarily formed through the oxidative polymerization of indole derivatives, such as 5,6-dihydroxyindole and 5,6-dihydroxyindole-2-carboxylic acid. It forms nanoscale particle aggregates [18]. Previous studies have associated melanins with antioxidant, metal-binding, photoprotective, and redox-regulating activities [19,20]. Melanin-based and melanin-inspired materials have also been investigated for wound healing, tissue repair, drug delivery, and neural tissue engineering [21,22]. These findings highlight the broad biomedical potential of melanin-related materials. However, they do not establish the systemic hemostatic activity of orally administered cuttlefish melanin.

More importantly, the relationship among the composition of high-purity cuttlefish melanin (HCM), its gastrointestinal transformation, and its systemic hemostatic activity has not yet been experimentally established. This issue is particularly important because CM is a macromolecular pigment whose biological activity after oral administration may depend on both the native material and its gastrointestinal transformation products. The small intestine is a major site of digestion, absorption, and biotransformation of dietary compounds; therefore, metabolites generated there may contribute to the hemostatic effects of CM. We hypothesized that HCM-derived metabolites in the small intestine may contribute to its hemostatic activity. To investigate this possibility, we first isolated and purified CM to obtain high-purity CM. We then performed two complementary experiments. Untargeted and targeted metabolomics based on UHPLC-Q-Orbitrap HRMS were used to characterize HCM-related metabolites in the small intestine. In parallel, a rat tail-bleeding model was used to evaluate hemostatic efficacy and safety by measuring bleeding volume, hemostasis time, and coagulation parameters. This study provides mechanistic insight into CM-associated hemostasis and its potential application as a marine-derived functional food ingredient.

## 2. Results

### 2.1. HCMs Are Natural Black Nanoparticles

**Figure 1:**
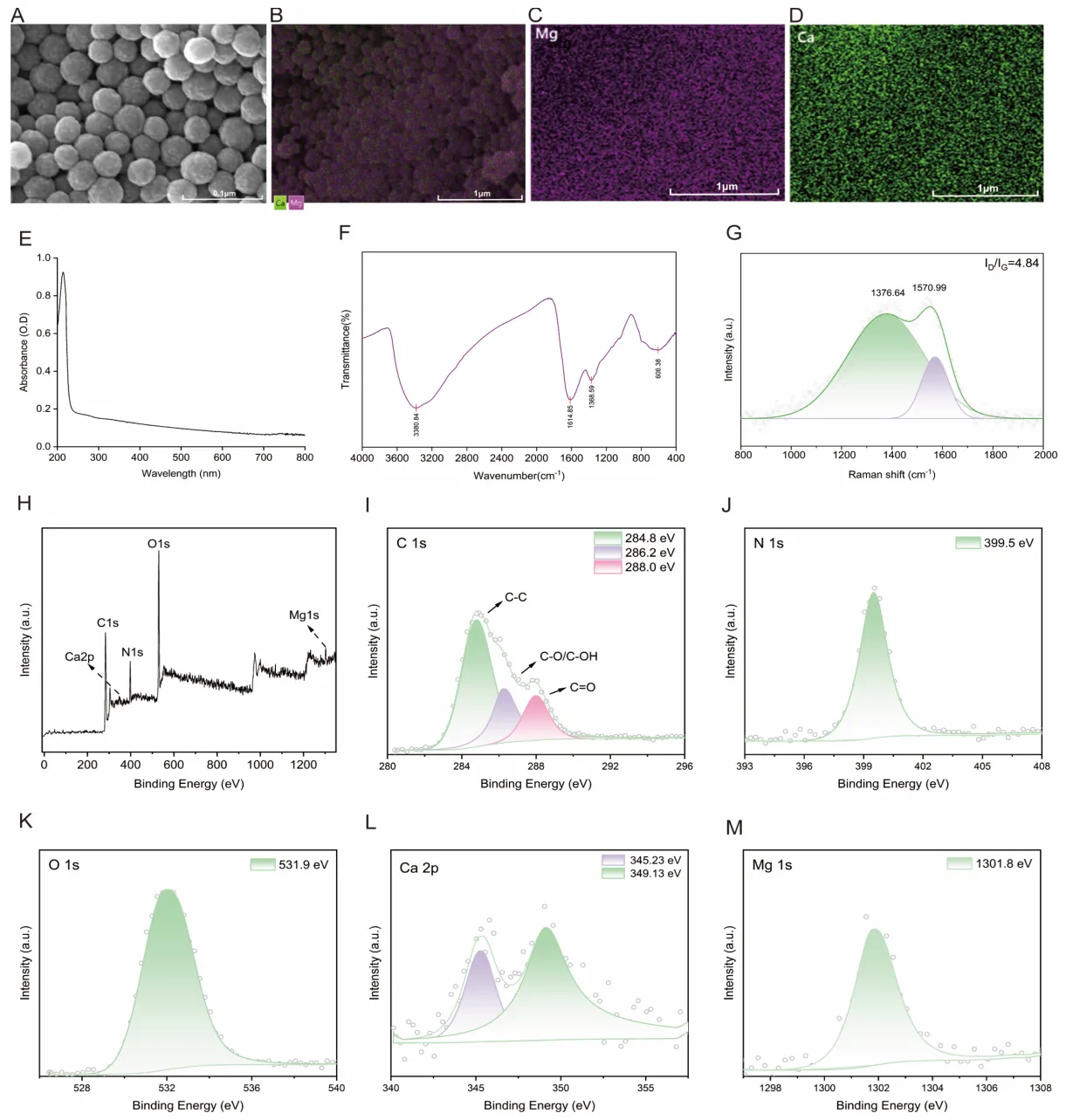
Spectroscopic characterization of squid ink melanin. (**A**) SEM micrographs of HCM, with scale bars representing 0.1 μm. (**B**) SEM-EDS elemental mapping of HCM (scale bars: 1 μm). (**C**) EDS map of Mg (fuchsia). (**D**) EDS map of Ca (green), (**E**) UV–Vis spectrum of HCM; (**F**) FTIR spectrum of HCM; (**G**) Raman spectrum. The combined analyses confirm the identity of the purified pigment as eumelanin with characteristic functional groups, optical absorption, elemental composition, and a highly disordered carbon structure (ID/IG = 4.85). (**H**) XPS survey spectrum of HCM and high-resolution (**I**) C1s, (**J**) N1s, (**K**) O1s, (**L**) Ca 2p, and (**M**) Mg 1s spectra.

The microstructure of HCM was examined using scanning electron microscopy (SEM) with energy-dispersive X-ray spectroscopy (EDS) analysis (Figure 1A–D). HCM is uniformly dispersed and naturally black, with a particle size between 100 and 200 nm. The primary metal elements in HCM were Mg and Ca, with total contents of 43.86 ± 0.97 mg/g and 30.20 ± 0.64 mg/g determined by inductively coupled plasma optical emission spectrometry (ICP-OES), respectively.

The ultraviolet–visible (UV–Vis) absorption spectrum of HCM is shown in Figure 1E. The spectrum exhibited a characteristic broad absorption band with a maximum at approximately 210 nm. The absorption intensity gradually decreased with increasing wavelength from the UV region to the visible region (400–800 nm), without any distinct absorption peaks in the visible range.

The FTIR spectrum of HCM is shown in Figure 1F. The spectrum exhibited a broad absorption band at approximately 3380 cm^−1^, attributed to the stretching vibrations of O–H and N–H groups, as well as intermolecular hydrogen bonding. The absorption peak at 1614 cm^−1^ corresponds to the stretching vibrations of C=C aromatic rings and C=O groups, which are characteristic of the indole structure of eumelanin. The peak at 1388 cm^−1^ is assigned to C–N stretching vibrations and COO– groups.

The Raman spectrum of HCM is shown in Figure 1G. The spectrum exhibited two characteristic broad bands centered at approximately 1376 cm^−1^ and 1570 cm^−1^, assigned to the D-band (disordered/sp^3^ carbon) and G-band (graphitic/sp^2^ carbon) of carbon-based materials, respectively. The ID/IG intensity ratio was calculated to be 4.85, indicating a predominantly disordered structure with abundant sp^3^-hybridized carbon centers and structural defects.

The surface elemental composition and chemical states of HCM were analyzed using XPS. The survey spectrum revealed three major peaks corresponding to C1s (~284.8 eV), O1s (~532.5 eV), and N1s (~399.8 eV), with relative atomic percentages of approximately 60.3%, 27.7%, and 12.0%, respectively (Figure 1H). The high-resolution C1s spectrum was deconvoluted into three peaks at 284.8 eV, 286.2 eV, and 288.0 eV, assigned to C–C, C–O, and C=O, respectively (Figure 1I). The N 1s spectrum exhibited a dominant peak at approximately 399.5 eV (Figure 1J), whereas the O 1s spectrum showed a major peak at approximately 531.9 eV (Figure 1K). In addition, distinct Ca 2p (~349.13 eV) (Figure 1L) and Mg 1s (~1301.80 eV) (Figure 1M) signals were detected on the melanin surface, with atomic percentages of 1.51% and 1.39%, respectively, confirming the adsorption of Ca and Mg species.

### 2.2. HCMs Are Converted into Amino Acids and Their Structural Analogs in the Small Intestine

H-HCM was dissolved in 10 mL of normal saline and administered via intubation into the stomachs of the rats. Two hours later, the rats were euthanized. Black nanoparticles were found in the stomach and a small amount in the duodenum. No black nanoparticles were detected in the jejunum or ileum. Therefore, we analyzed the metabolic products from the middle parts of the small intestine (jejunum and ileum) two hours after H-HCM was administered into the stomachs of the rats.

**Figure 2:**
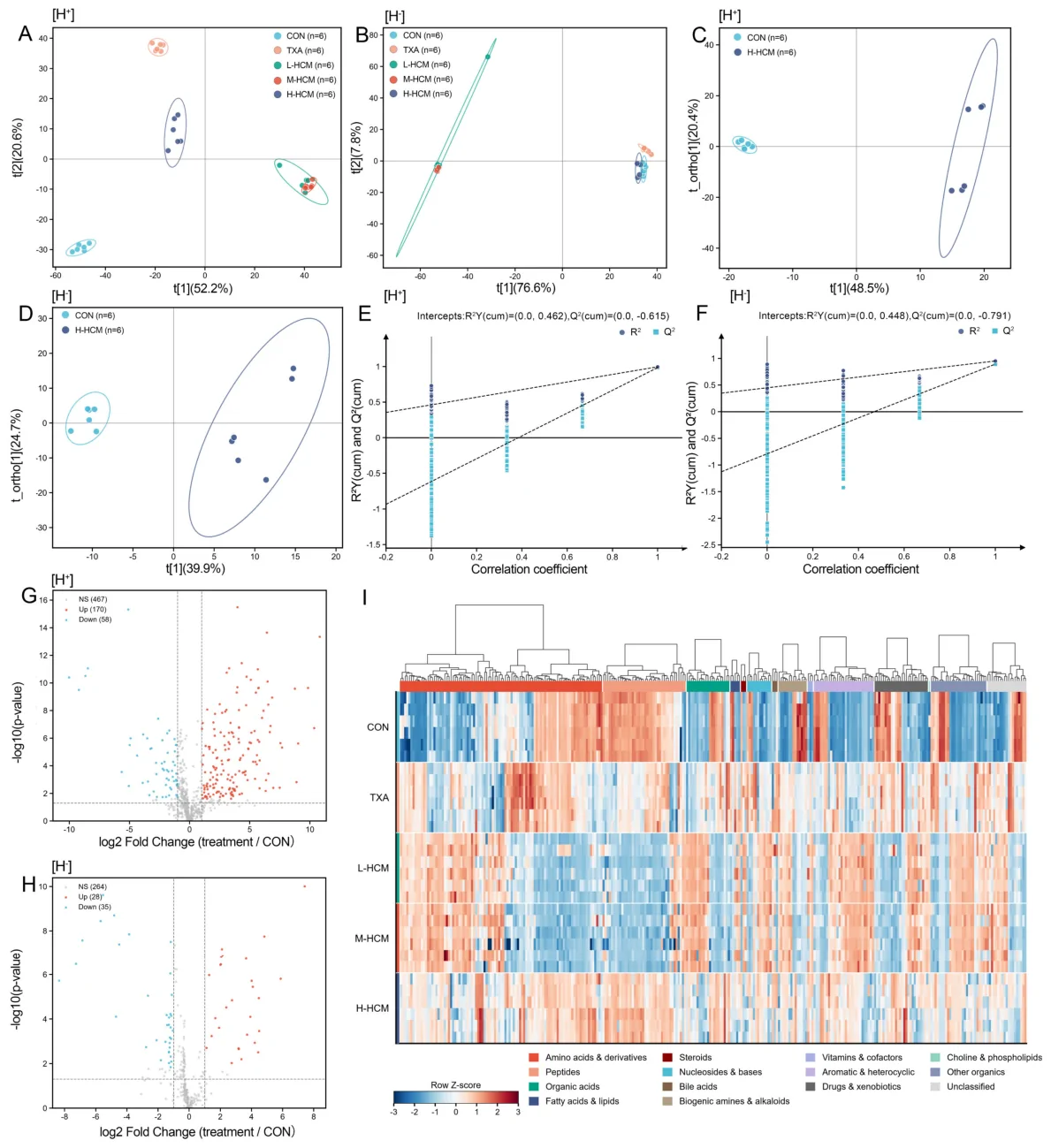
Untargeted metabolomic profiles of the CON, TXA, L-HCM, M-HCM, and H-HCM groups. (**A**,**B**) PCA score plots in positive and negative ion modes, respectively. (**C**,**D**) OPLS-DA score plots for CON versus H-HCM in positive and negative ion modes, respectively. (**E**,**F**) Corresponding permutation tests, with R^2^Y and Q^2^ intercepts indicated. (**G**,**H**) Volcano plots of differential metabolites between the treatment and CON groups in positive and negative ion modes. (**I**) Hierarchical clustering heatmap of differential metabolites across the five groups in positive ion mode. CON: control group; TXA: positive administration group; L-HCM: low-dose group; M-HCM: medium-dose group; H-HCM: high-dose group.

The untargeted metabolomics analysis of the CON, L-HCM, M-HCM, TXA, and H-HCM groups was performed using UHPLC-Q-Orbitrap HRMS with Full MS and Full MS/dd–MS^2^ scanning modes, and the positive and negative ion modes were detected, respectively. First, the Principal Component Analysis (PCA) score plots (Figure 2A,B) revealed clear group-dependent separation in the positive-ion mode ([H]^+^) among the CON, TXA, and different HCM intervention groups. In the negative-ion mode ([H]^−^), the L-HCM group showed a distinct separation pattern, whereas the other groups were generally clustered within relatively similar regions. To further characterize the metabolic alterations induced by H-HCM intervention, supervised orthogonal partial least squares discriminant analysis (OPLS-DA) was performed to compare the CON and H-HCM groups in both ionization modes (Figure 2C,D). The score plots showed clear and complete separation between the two groups. The first predictive component, t [1], explained 48.5% and 39.9% of the model variation in the positive- and negative-ion modes, respectively.

Permutation test (Figure 2E,F) yielded Q^2^ regression-line intercepts of −0.615 and −0.791 in the positive- and negative-ion modes, respectively. Both intercepts were below zero, supporting the robustness of the OPLS-DA models and indicating that the data meet the requirements for interpretability and predictability of the analysis. Overall, H-HCM intervention markedly altered the small-intestinal metabolic profile. The volcano plots further demonstrated extensive metabolic alterations in the H-HCM group compared with the CON group. In the positive-ion mode, 170 metabolites were upregulated, and 58 were downregulated, whereas 467 features showed no significant differences. In the negative-ion mode, 28 metabolites were upregulated, and 35 were downregulated, while 264 features were not significantly changed (Figure 2G,H). The hierarchical clustering heatmaps showed distinct metabolite expression patterns between the CON and H-HCM groups in both positive (Figure 2I) and negative ion modes (Figure S1), with amino acids and their derivatives representing an important metabolite category among the discriminating features. Taken together, the untargeted metabolomics results demonstrated that H-HCM intervention markedly altered the metabolic composition of the small intestine and highlighted amino acid-related metabolites as a potentially important metabolic signature. Accordingly, subsequent analyses focused on targeted quantitative profiling of amino acids to validate and further characterize the amino acid metabolic changes associated with H-HCM intervention.

**Figure 3:**
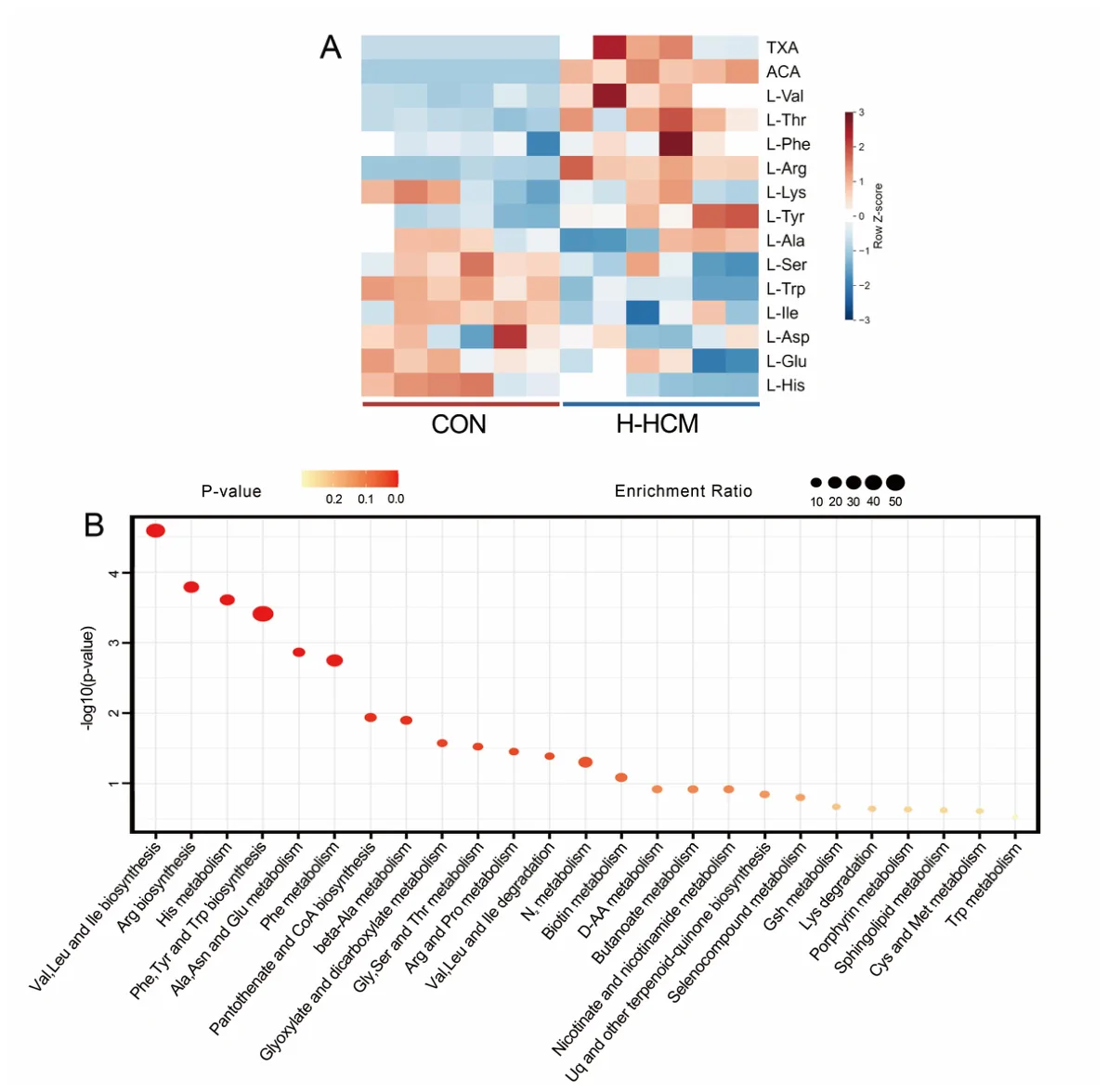
Targeted metabolomics statistics of CON and H-HCM. (**A**) Heatmap of hierarchical clustering analysis by positive and negative ion scanning mode. (**B**) Summary of enrichment analysis and pathway analysis using MetaboAnalyst 5.0. Enrichment analysis of CON vs. H-HCM. CON: control group; TXA: positive administration group; H-HCM: high-dose group; ACA: 6-Aminocaproic acid; Val: Valine; Thr: Threonine; Phe: Phenylalanine; Arg: Arginine; Lys: Lysine; Tyr: Tyrosine; Ala: Alanine; Ser: Serine; Try: Tryptophan; Ile: Isoleucine; Asp: Aspartic acid; Glu: Glutamate; His: Histidine.

Based on the results of untargeted metabolomics analysis, we found that the differences in intestinal metabolites between the CON and H-HCM groups are primarily due to nitrogen-containing organic compounds. To ensure the accuracy of the data, we conducted targeted metabolomics analysis on these compounds utilizing PRM scanning mode. We listed the positive and negative ions of the compounds for identification using the molecular ion peak and three fragment ion peaks, and then validated the identifications with standards. The peak areas of differential compounds between the CON and H-HCM groups were collected for the heatmap. As shown in Supplement Table S1 and Figure 3A, the heatmap displays the cluster analysis of differential metabolites. TXA and 6-aminocaproic acid (ACA) were detected in the small intestine following HCM administration, along with significant upregulation of valine, threonine, phenylalanine, tyrosine, alanine, aspartic acid, and arginine, and considerable downregulation of serine, isoleucine, tryptophan, lysine, glutamate, and histidine (Table S2).

The significant metabolites, amino acids, and their structural analogs were then mapped onto biochemical pathways using metabolic pathway and enrichment analyses. We conducted KEGG pathway analysis on the MetaboAnalyst platform, identifying 25 metabolic pathways in the small intestine treated with CON and H-HCM (Figure 3B). The six pathways with the highest differential metabolite enrichment included: (1) Valine, leucine, and isoleucine biosynthesis; (2) Arginine biosynthesis; (3) Histidine metabolism; (4) Biosynthesis of phenylalanine, tyrosine, and tryptophan; (5) Alanine, aspartate, and glutamate metabolism; and (6) Phenylalanine metabolism.

### 2.3. Oral Administration of HCM in Rats Effectively Reduces Hemorrhage Volume and Hemostasis Time

**Figure 4:**
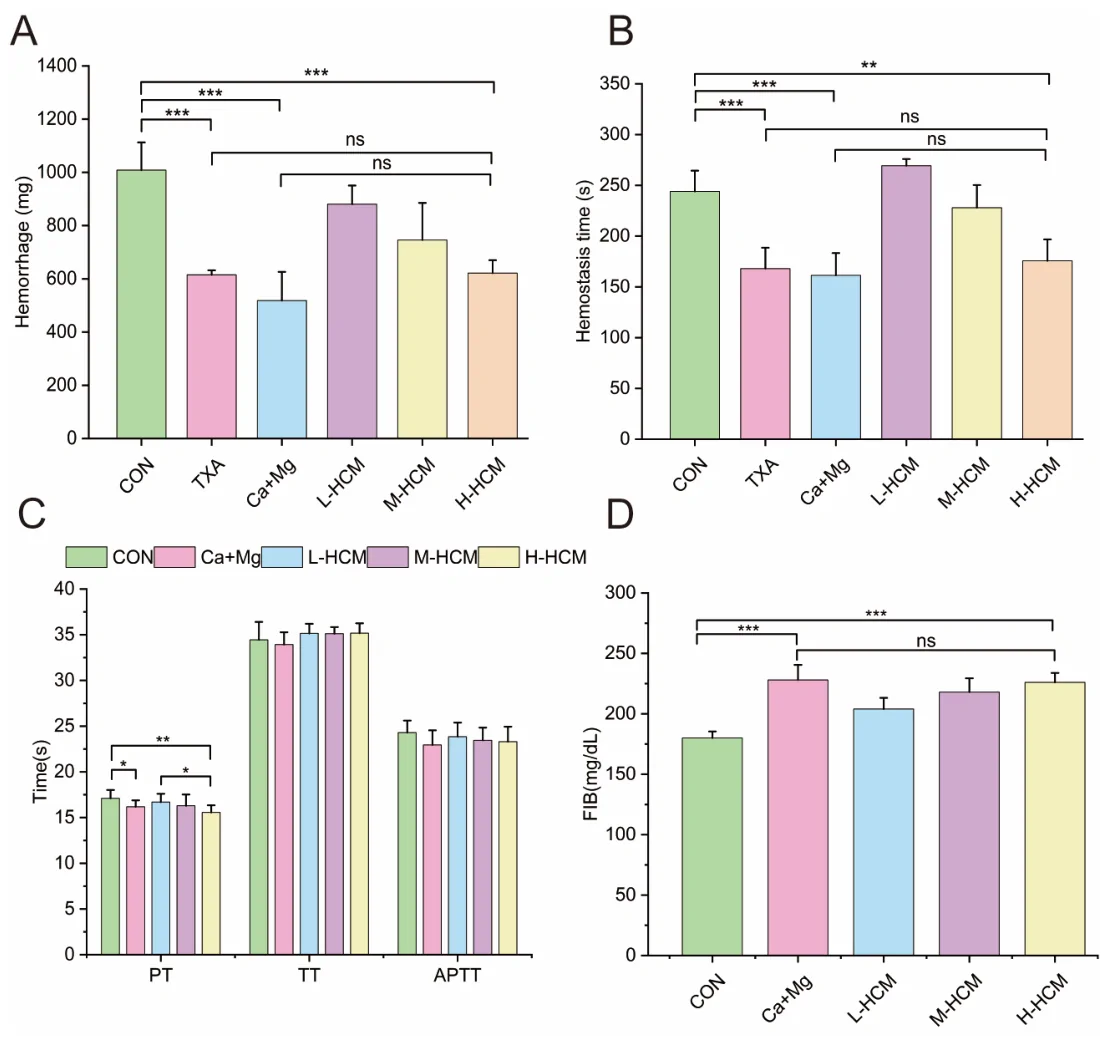
Coagulation profile 2 h after gavage HCM administration in rats. The bar chart in (**A**) shows hemorrhage volume, and (**B**) displays hemostasis time for CON, TXA, Ca+Mg, and three doses of HCM. The bar chart in (**C**) presents PT, APTT, and TT, while (**D**) illustrates FIB levels for CON, Ca+Mg, and three doses of HCM. Statistical significance is as follows: ns: *p* > 0.05, * *p* < 0.05, ** *p* < 0.01, *** *p* < 0.001, *n* = 6.

**Figure 5:**
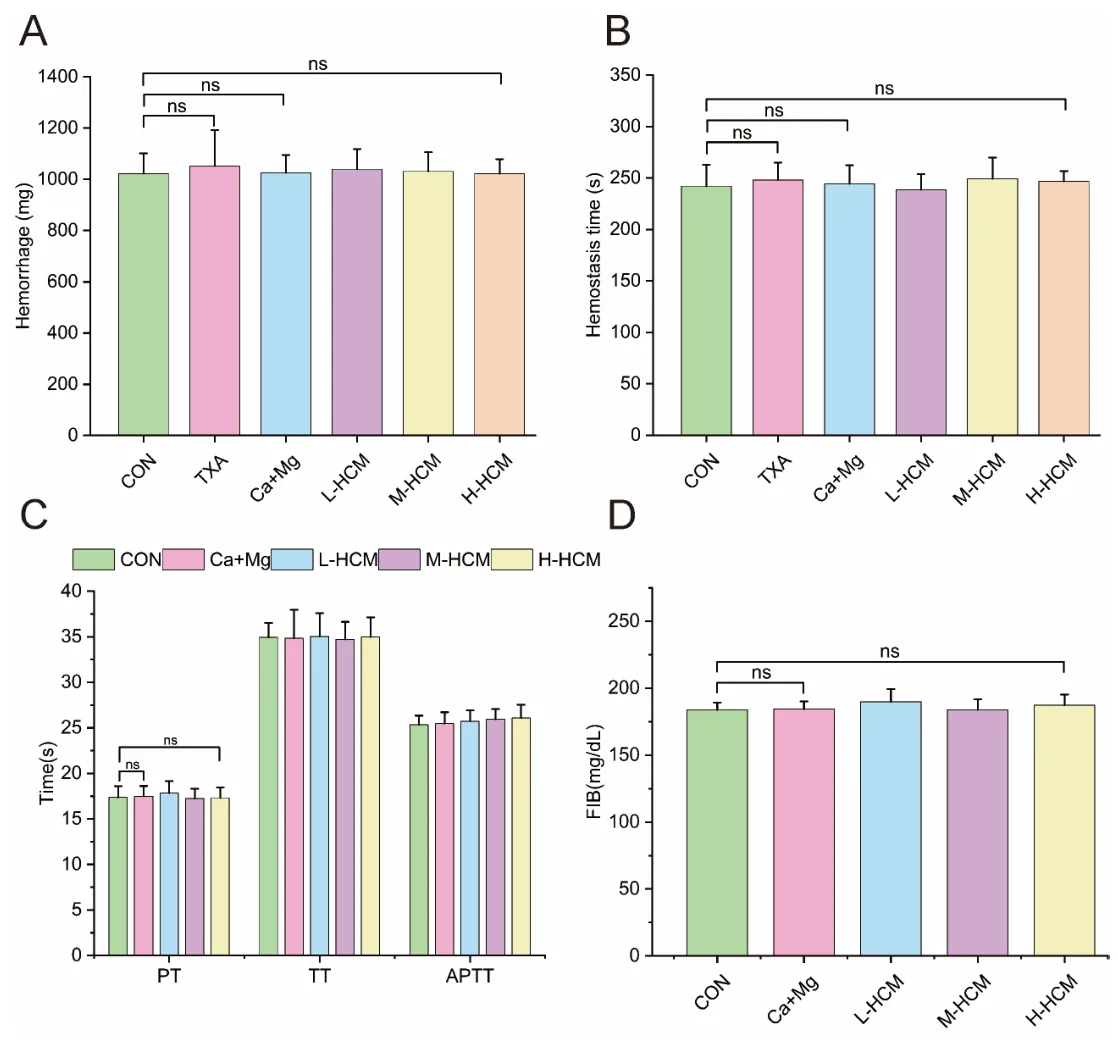
Coagulation profile 24 h after gavage HCM administration in rats. The bar chart in (**A**) shows hemorrhage volume, and (**B**) displays hemostasis time for CON, TXA, Ca+Mg, and three doses of HCM. The bar chart in (**C**) presents PT, APTT, and TT, while (**D**) illustrates FIB levels for CON, Ca+Mg, and three doses of HCM. Statistical significance is as follows: ns: *p* > 0.05, *n*= 6.

The hemorrhage volume in rats administered TXA, Ca+Mg, and three doses of HCM was lower than that of CON after 2 h (Figure 4A). TXA (615 ± 17 mg), Ca+Mg (518 ± 107 mg), and H-HCM (612 ± 49 mg) show highly significant differences compared to CON (1008 ± 104 mg) (*p* < 0.001). There was no significant difference between TXA (615 ± 17 mg) and H-HCM (612 ± 49 mg) (*p* > 0.05), or between Ca + Mg (518 ± 107 mg) and H-HCM (612 ± 49 mg) (*p* > 0.05). With increasing doses of HCM, hemorrhage volume decreased in a dose-dependent manner. These results suggest that all three doses of HCM effectively reduce latent blood loss in rats. However, the hemorrhage volume in rats 24 h after administration showed no significant difference compared with the CON group (Figure 5).

The hemostasis time in rats administered TXA, Ca+Mg, M-HCM, and H-HCM was also lower than in the CON group after 2 h (Figure 4B). TXA (168 ± 21 s) differed extremely significantly from the CON group (244 ± 21 s) (95% CI: −110.9 to −41.1 s, Cohen’s d = 3.62, *p* < 0.001). Ca+Mg (161 ± 22 s) also differed extremely significantly from the CON group (95% CI: −117.6 to −47.8 s, Cohen’s d = 3.88, *p* < 0.001). H-HCM (176 ± 22 s) also shows a considerable difference compared to the CON group (244 ± 21) (*p* < 0.01). However, no significant difference was observed between TXA (168 ± 21 s) and H-HCM (176 ± 22 s) (*p* > 0.05), or between Ca+Mg (161 ± 22 s) and H-HCM (176 ± 22 s) (*p* > 0.05). With increasing doses of HCM, hemostasis time decreased in a dose-dependent manner. These results suggest that M-HCM and M-HCM effectively reduce latent blood loss in rats. However, the hemostasis time in rats 24 h after administration showed no significant difference compared with the CON group (Figure 5).

The hemorrhage volume and hemostasis time of H-HCM were not significantly different from those of the first positive control group (TXA) and the second positive control group (Ca+Mg). In conclusion, HCM is an effective nano-hemostatic agent.

### 2.4. The Safety of HCM Is Evaluated Using Four Coagulation Tests

The present study investigates the impact of Ca^2+^ and Mg^2+^, released by HCM in the intestine, on plasma coagulation parameters, including PT, APTT, TT, and FIB levels. These four parameters are critical for assessing the efficiency of the clotting pathways in plasma. Specifically, APTT and PT were used to evaluate the intrinsic and extrinsic clotting pathways. A significant shortening of APTT and PT activates both the endogenous and exogenous clotting pathways. In contrast, TT and FIB reflect the third stage of the coagulation process. A shortening of TT or an increase in FIB levels indicates the activation of thrombin-mediated fibrin formation. PT, APTT, TT, and FIB are key clinical coagulation function screening parameters. They are also important indicators in laboratory research and the safety evaluation of hemostatic or anticoagulant drugs. We assessed the hemostatic pathway and safety of HCM by measuring PT, APTT, TT, and FIB. Two hours after administration, the PT values in the Ca+Mg and three HCM dose groups were lower than those in the CON group. The PT value in the Ca+Mg group (16.2 ± 0.7 s) was significantly lower than that in the CON group (17.1 ± 0.9 s) (95% CI: -1.97 to -0.12 s, Cohen’s d = 1.12, *p* < 0.05). The PT value in the H-HCM group (15.6 ± 0.8 s) was also lower than that in the CON group (17.1 ± 0.9 s) (*p* < 0.01). The APTT values in the Ca+Mg and three HCM dose groups were lower than those in the CON group; however, none of these differences were statistically significant (*p* > 0.05). The TT values in the Ca+Mg and three HCM dose groups were not different from those in the CON group (*p* > 0.05) (Figure 4C). The FIB values in the Ca+Mg and three HCM dose groups were higher than those in the CON group. The FIB values in the Ca+Mg group (228 ± 13 mg/dL) and the H-HCM group (226 ± 8 mg/dL) were substantially higher than those in the CON group (180 ± 5 mg/dL) (*p* < 0.001). There was no difference between the Ca+Mg group (228 ± 13 mg/dL) and the H-HCM group (226 ± 8 mg/dL) (*p* > 0.05). These data indicate that the HCM and Ca+Mg groups achieve hemostasis through the extrinsic pathway (Figure 4D). Twenty-four hours after administration, there were no significant differences in APTT, TT, FIB, and PT among the groups of rats (Figure 5).

## 3. Discussion

### 3.1. HCM Is a Natural, Black, Spherical-Shaped Nanoparticle

It forms when DHICA and DHI combine with Mg^2+^ and Ca^2+^. In this structure, Mg^2+^ and Ca^2+^ create coordinate covalent bonds. These bonds connect to the phenol hydroxyl groups or carboxyl groups on DHICA and DHI. Small changes in eumelanin’s building blocks (monomers) can alter its biological effects. These changes depend mainly on the decarboxylation of L-dopachrome. This decarboxylation step is a key branch point in eumelanin’s biosynthesis pathway [23]. As a result, the DHICA/DHI ratio in natural eumelanin significantly affects the biological activity and the morphology of the natural eumelanin granules [19,23,24].

The UV–Vis absorption spectrum exhibited a featureless broad absorption profile with a maximum at approximately 210 nm, which is characteristic of natural melanin [25,26]. The gradual decrease in absorbance with increasing wavelength toward the visible region is attributed to the complex conjugated structure of melanin, which absorbs light across a wide range of wavelengths. The absence of sharp peaks in the visible region confirms the removal of low-molecular-weight chromophoric impurities during the purification process.

The FTIR spectrum revealed characteristic absorption bands typical of melanin, including O–H/N–H stretching (~3380 cm^−1^), C=C/C=O stretching (~1614 cm^−1^), and C–N/COO^−^ stretching (~1368 cm^−1^) [25]. These functional groups are consistent with the DHI/DHICA-based structure of melanin. The broad and overlapping nature of these bands reflects the heterogeneous and cross-linked structure of the melanin polymer.

The Raman spectrum exhibited two characteristic broad bands at approximately 1376 cm^−1^ (D-band) and 1570 cm^−1^ (G-band), which are consistent with the typical Raman spectral features of carbonaceous materials and melanin pigments [26]. The D-band arises from disordered sp^3^-hybridized carbon domains, while the G-band arises from ordered sp^2^-hybridized aromatic carbon domains. The presence of both bands reflects the heterogeneous structure of melanin, which contains both graphitic-like ordered domains and amorphous disordered regions within the polymer network [27]. The high ID/IG ratio (4.85) observed in HCM provides quantitative evidence for its structurally disordered nature. This high degree of disorder can be attributed to several factors: (1) the heterogeneous polymerization of DHI and DHICA monomers through multiple connectivity sites, leading to a variety of local structures, and (2) the presence of cross-linking and branching within the polymer network.

XPS analysis confirmed that HCM is composed primarily of carbon, oxygen, and nitrogen, the principal elements associated with the DHI/DHICA-derived polymeric structure of eumelanin [26]. The prominent N 1s signal at approximately 399.5 eV is particularly noteworthy because it indicates the presence of indole-derived nitrogen species, including pyrrolic and amine/amide-like nitrogen, which are characteristic of the melanin backbone [25]. The relatively high oxygen content (approximately 27.7 at. %) suggests that the HCM surface contains abundant oxygen-bearing functional groups, including hydroxyl, carbonyl, and carboxyl groups, consistent with the FTIR results. Deconvolution of the high-resolution C 1s spectrum revealed three components centered at 284.8, 286.2, and 288.0 eV, which were assigned to C-C, C-O, and C=O bonds, respectively. These results indicate that the HCM surface is enriched in aromatic carbon and oxygen-containing functional groups.

In addition to the characteristic signals of the melanin matrix, distinct Ca 2p and Mg 1s peaks were detected at approximately 349.13 and 1301.80 eV, respectively. The corresponding atomic percentages of Ca and Mg were 1.51% and 1.39%, confirming the presence of these metal species on the HCM surface. Therefore, the coexistence of the Ca and Mg signals with the C 1s, N 1s, and O 1s signals of the melanin matrix suggests that Ca^2+^ and Mg^2+^ may interact with surface carboxyl groups, potentially through ionic interactions or surface complexation, rather than being present solely as a simple physical mixture or a separately doped phase. Nevertheless, because XPS alone cannot definitively establish specific metal–ligand binding modes, this interpretation should be considered evidence of a possible association between Ca^2+^/Mg^2+^ and the oxygen-containing functional groups on the HCM surface [28].

### 3.2. The Ca and Mg Released by HCM in the Intestine May Exert a Hemostatic Effect by Activating Coagulation Factors

After gastrointestinal digestion, HCM nanoparticles release Ca and Mg in the intestine, which may contribute to the regulation of the coagulation system [29].

**Figure 6:**
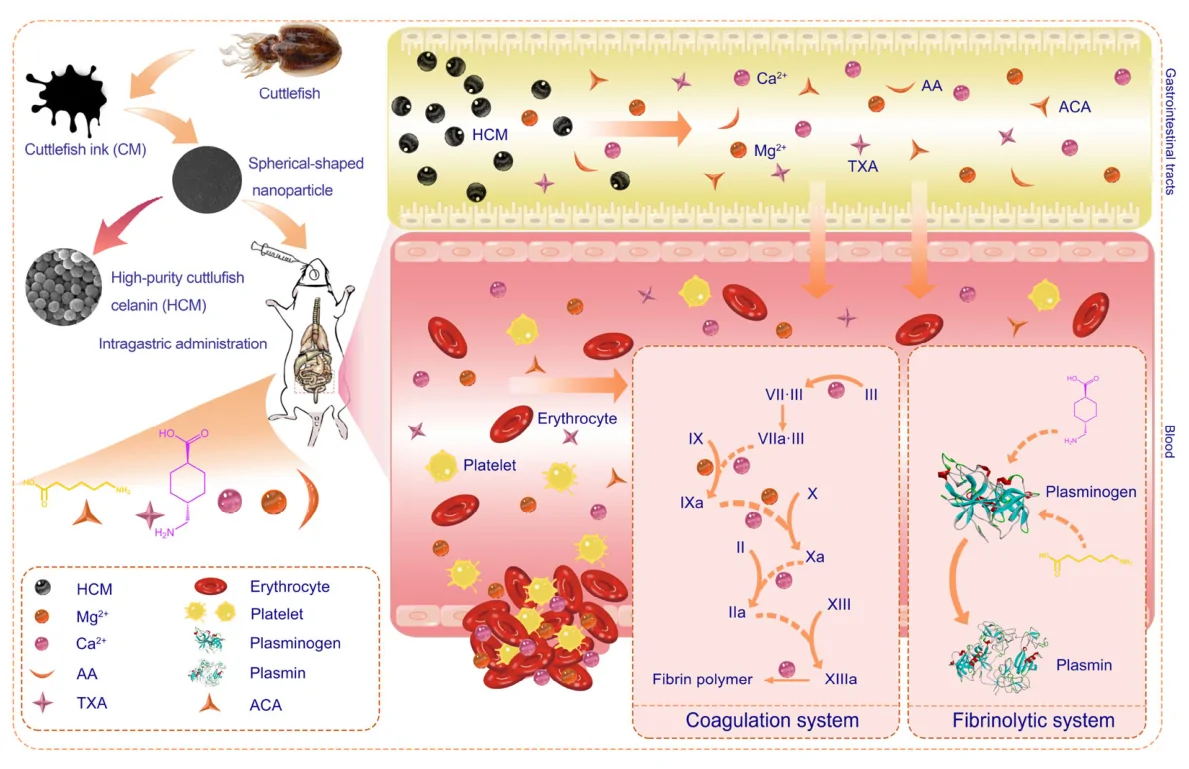
Proposed hemostatic mechanism of HCM. The Ca and Mg released by HCM in the intestine exert a hemostatic effect by activating coagulation factors in the coagulation system. HCM is metabolized into TXA and ACA in the intestine, inhibiting plasmin and plasminogen, which facilitates hemostasis. HCM: high-purity cuttlefish melanin; TXA: tranexamic acid; AA: amino acids; ACA: 6-Aminocaproic acid. The rat schematic used in this study is based on an image from our prior publication [36].

Numerous clinical studies have confirmed the hemostatic properties of Mg^2+^ [29,30]. Both Ca^2+^ and Mg^2+^ play key roles in stabilizing the conformation and enhancing the activity of factor IX. Additionally, Mg^2+^ facilitates the activation of factor X by factor IX in the presence of factor VIII, phospholipids, and Ca^2+^. Another crucial function of Mg^2+^ is its promotion of factor IX activation by the factor VII–tissue factor complex, a key initiator of blood coagulation in vivo (Figure 6) [31].

Following endothelial injury, subendothelial collagen is exposed. This exposure activates platelets, triggering the intrinsic coagulation pathway. The damaged vascular tissue releases tissue factor (TF), which activates the extrinsic coagulation pathway. Thrombin is rapidly generated at the site of injury. Platelets flowing through the injured vessel adhere to the collagen fibers at the site of damage. In the liver, the glutamic acid domains of clotting factors II, VII, IX, and X are modified with the help of vitamin K, and the action of γ-glutamate carboxylase converts these glutamic acid domains into γ-carboxyglutamate (Gla) domains [29,32,33]. This modification imparts a negative charge to the clotting factors. The cell membrane also carries a negative charge. Ca^2+^ and Mg^2+^ bridge the clotting factors and the cell membrane, thus initiating coagulation. Either intrinsic or extrinsic coagulation pathways rely on Ca^2+^ and Mg^2+^ to position factors II, VII, IX, and X on the injured cell membrane (Figure 6) [34,35].

### 3.3. TXA and ACA Were Detected in the Intestine After HCM Administration, Which Inhibits Plasmin and Plasminogen, Thereby Facilitating Hemostasis

Following HCM administration, TXA and ACA were detected in the small intestine; however, whether they are converted from HCM, and by what pathway, remains unclear. Both ACA and TXA are lysine derivatives widely used in clinical practice to reduce surgical bleeding and treat hyperfibrinolysis. These compounds bind to lysine-binding sites on plasminogen and plasmin, competing with lysine residues on fibrin. This competitive interaction inhibits the binding of plasminogen to fibrin, thereby preventing the formation of plasmin. Furthermore, at elevated concentrations, both ACA and TXA can directly associate with plasmin, suppressing its activity and blocking plasmin generation or its degradation of fibrin.

### 3.4. HCM Is a Safe and Effective Nanoparticle Hemostatic Agent

The tail-bleeding assay demonstrated that HCM significantly reduced both blood loss and bleeding time, confirming its potent hemostatic efficacy. To elucidate the underlying mechanisms and evaluate safety, we analyzed coagulation parameters. Compared with the CON group, HCM administration significantly shortened PT and significantly increased FIB levels, whereas APTT and TT showed no significant changes.

To assess the physiological and safety implications of these changes, we compared our measured values with established reference intervals for rats [37]: PT 13.32–17.37 s, APTT 10.54–29.90 s, TT 16.08–58.20 s, and FIB 180–504 mg/dL. In the CON group, PT (17.1 ± 0.9 s) approached the upper bound of the normal range, while FIB (180 ± 5 mg/dL) was at the lower bound. Following HCM administration, PT shortened to 15.6 ± 0.8 s, and FIB increased to 226 ± 8 mg/dL. Importantly, although these changes were statistically significant relative to the control, all measured values remained well within the established physiological reference intervals.

The marked shortening of PT, without concurrent changes in APTT or TT, suggests that HCM primarily enhances the extrinsic coagulation pathway, which is calcium-dependent and mechanistically consistent with Ca released from HCM acting as a cofactor for the tissue factor–factor VIIa complex. Concurrently, the elevated FIB levels indicate increased fibrinogen availability for thrombin-mediated fibrin polymerization. The directional shift—PT moving away from the upper bound and FIB moving away from the lower bound toward the mid-range of normal—indicates a physiologically meaningful procoagulant shift for hemostasis, but does not constitute a hypercoagulable state exceeding normal limits. The transient nature of these effects, resolving by 24 h post-administration, further supports the safety profile of HCM.

This study has several limitations. First, the open-label design was necessitated by the obvious color differences between the CON and HCM gavage solutions, which precluded blinding of the investigators during the experiment. However, to minimize potential bias, statistical analysis was performed by an investigator blinded to group allocation. Second, the study was conducted in rats, and while this model is well-established for hemostasis research, it may not fully recapitulate human physiology. Third, while we observed correlations between HCM administration and coagulation changes, the precise causal relationships require further validation through assays such as thrombin generation tests, platelet function analysis, and protein expression studies. Therefore, although our findings suggest that HCM exhibits promising hemostatic activity with an acceptable safety margin in this model, further validation in other preclinical models and ultimately in clinical studies is warranted.

## 4. Materials and Methods

### 4.1. Chemical Reagents

Standards of tranexamic acid (TXA, CAS: 1197-18-8), 6-aminocaproic acid (ACA, CAS: 60-32-2) (purity ≥ 98%), and alkaline protease (200 units/mg) were purchased from Shanghai Yuanye Biotechnology Co., Ltd. (Shanghai, China). A mixture of the twenty standard amino acids was purchased from the National Institute of Metrology (Beijing, China). UPLC-MS-grade methanol, acetonitrile, and formic acid used for UHPLC-Q-Orbitrap HRMS analysis were obtained from Thermo Fisher Scientific Inc. (Waltham, MA, USA). PT kit, TT kit, APTT kit, FIB kit, and buffer fluid were purchased from Beijing Biochem Xinji S&T Development Co., Ltd. (Beijing, China). Calcium gluconate and magnesium gluconate were purchased from Qingdao Hanghaike Co., Ltd. (Qingdao, China).

### 4.2. Melanin Purification

Frozen cuttlefish were bought from Qingdao Hanghaike Co., Ltd. (Qingdao, China), thawed to collect cuttlefish sepia ink, and stored at 20 °C. Melanin was extracted following the method reported in the literature with slight modifications [38,39]. Twenty times the volume of distilled water was added to the thawed ink, and it was stirred at 4 °C for 12 h. The diluted ink was centrifuged in a TGL-20 bR centrifuge (Anting Co. Ltd., Shanghai, China) for 15 min (8944 g, 4 °C) to remove the supernatant. This process was repeated six times to remove the water-soluble impurities in the ink. After the precipitate was collected, it was dried by vacuum freeze-drying. Petroleum ether degreasing of the dry sediment was repeated three times using the Soxhlet extraction method, and fat-free melanin was obtained.

The alkaline protease was used for enzymatic hydrolysis. The enzyme-to-melanin ratio was 1:50 (W: W), and the reaction was performed at 50 °C and pH 10 for 8 h. The reaction was stopped by incubating the solution at 95 °C for 15 min. The resulting solution was centrifuged for 15 min (8944 g, 4 °C), and after vacuum freeze-drying, the enzymatic melanin was obtained. The enzymatic melanin powder was then immersed in an 80% ethanol solution (material: liquid-1:25), ultrasonicated at 90 Hz for 30 min, and centrifuged at 8944 g and 4 °C for 10 min. The supernatant was then discarded. The insoluble material was rewashed with 80% ethanol and centrifuged as described previously. The extraction process was repeated three times, and the sediment was collected and vacuum-dried, resulting in high-purity CM.

### 4.3. Structural Characterization of HCM

#### 4.3.1. SEM-EDS Determined Microstructure Surface Morphology and Elemental Composition of HCM

The microstructure, surface morphology, and elemental composition of the HCM were analyzed using SEM-EDS, following the protocol with slight modifications [40,41]. The HCM was coated with a thin layer of gold to prevent charging during imaging. The HCMs were assessed using an JEOL JSM-IT800 SEM (JEOL Ltd., Tokyo, Japan) equipped with an EDS (Ultim MAX 65, Oxford Instruments, Oxford, UK), operating at an accelerating voltage of 20 kV. Images of the HCM were captured at magnifications of 50× and 150×, and a higher-resolution image of the HCM was selected. The elemental composition of the HCM was qualitatively assessed, and the elemental contents were semi-quantitatively analyzed using energy-dispersive spectroscopy.

#### 4.3.2. ICP-OES Determined Mineral Elements of HCM

The main metal elements in HCM were determined by PerkinElmer Optima 8000 ICP-OES (PerkinElmer, Waltham, MA, USA). Briefly, 0.1 g samples were digested with 5 mL nitric acid and 1 mL perchloric acid at 200 °C. The digest solution was diluted to 100 mL with distilled water before assaying. Calibration curves were constructed using standard solutions at concentrations of 0.1–10 mg/L, with correlation coefficients (r^2^) greater than 0.999. Parallel samples were analyzed, and the relative standard deviation (RSD) was below 5% for both Ca and Mg.

#### 4.3.3. FTIR Analysis of HCM

We freeze-dried the HCM and mixed it with dry KBr at a 1:100 ratio. The mixture was ground uniformly in an agate mortar and then pressed into a transparent pellet at 10 tons for 2 min. We recorded the FTIR spectrum using a Fourier transform infrared spectrometer (FTIR) (Thermo Fisher Scientific, Waltham, MA, USA). The spectrum was collected over the range of 4000–400 cm^−1^ with a resolution of 4 cm^−1^.

#### 4.3.4. UV–Vis Analysis of HCM

We recorded the UV–Vis spectrum of squid ink melanin using an Evolution 201 spectrophotometer (Thermo Fisher Scientific, Waltham, MA, USA). The scan covered 200–800 nm. We dissolved the HCM in 1% NaOH and placed it in a quartz cuvette (1 cm path length). We used 1% NaOH as the blank.

#### 4.3.5. Raman Spectroscopy Analysis of HCM

Raman spectrum of squid ink melanin was recorded using a Raman spectrometer (Renishaw, Wotton-under-Edge, UK). equipped with a 532 nm laser excitation source. The laser power was maintained at 150 mW to avoid sample degradation. The spectrum was collected over the wavenumber range of 800–2000 cm^−1^ with a spectral resolution of 1 cm^−1^. The acquisition time was 1 s per scan, and multiple scans were accumulated to improve the signal-to-noise ratio.

#### 4.3.6. XPS Analysis of HCM

XPS analysis was performed using an ESCALAB 250Xi spectrometer (Thermo Fisher Scientific, Waltham, MA, USA) with monochromated Al Kα radiation (hν = 1486.6 eV). The X-ray source was operated at 150 W, with survey spectra recorded at a pass energy of 200 eV and high-resolution spectra at 30 eV. The base pressure in the analysis chamber was maintained below 1.0 × 10^−9^ mbar. Survey spectra were acquired over a binding energy range of 0–1200 eV. High-resolution XPS spectra (C 1s, N 1s, O 1s, Ca 2p, and Mg 1s) were acquired to evaluate the chemical states of the constituent elements. Binding energies were calibrated against the adventitious carbon C 1s peak (284.8 eV). Peak fitting and data processing were carried out using Avantage 6.6.0 (Thermo Scientific, Waltham, MA, USA).

### 4.4. Animal Trials

One hundred and fourteen rats (Wistar, approximately 250 g body weight) and standard rat chow were purchased from Beijing Vital River Laboratory Animal Technology Co., Ltd. (Beijing, China). The Wistar rat was selected as the experimental model due to its well-characterized physiological stability and metabolic similarity to humans. The animals were housed in a room at 23 ± 1 °C and 60% humidity with a 12-h light–dark cycle and were provided with free access to water and a standard diet for one week before the experiment to adapt to the environment. The open-formula purified diets contained approximately 18% protein, 55% carbohydrate, and 4% fat by weight. Twelve hours of fasting with free access to water were required before the experiment. To minimize potential confounders, the order of treatments and measurements was randomized. Additionally, the location of cages within the animal facility was rotated regularly to control for environmental gradients. The animal procedures and care used in this study were approved by the Shaanxi University of Technology and the Technology Ethics Committee (Approval #2025012301).

This study comprised two group experiments to examine the intestinal metabolites of HCM and evaluate its coagulation effects using one hundred and fourteen male rats. Seventy-two rats were used to evaluate coagulation function at 2 and 24 h after gavage administration, and each group was divided into six subgroups (*n* = 6). We set the oral gavage dose according to the package insert of the clinical drug, Haimo hemostatic capsules. For the experimental group, 400 mg/kg (low-dose group, L-HCM), 800 mg/kg (medium-dose group, M-HCM), and 1200 mg/kg (high-dose group, H-HCM) (*n* = 6) of HCM dissolved in 5 mL normal saline were intubated into the stomachs of eighteen rats. Six rats were intubated with 100 mg/kg of TXA as the first positive administration group (TXA). Six rats were intubated with Mg ^2+^ (54 mg/kg) and Ca ^2+^ (40 mg/kg) as the second positive administration group (Ca+Mg). Six rats were intubated with 5 mL of normal saline solution and used as the control group (CON).

The HCM doses were calculated based on the package insert for Haimo Hemostatic Capsules. Each capsule contains 0.33 g of the formulation, and the recommended adult dosage is six capsules per administration, three times daily, assuming an adult body weight of 60 kg. After conversion based on body surface area, the clinically equivalent dose for rats was estimated to be approximately 611 mg/kg. This dose fell within the dose range selected for the present study, indicating that the experimental dose design appropriately covered a clinically relevant exposure level.

According to ICP-OES measurements, the Mg and Ca contents of HCM were 43.86 ± 0.97 mg/g and 30.20 ± 0.64 mg/g, respectively. Based on the administered HCM doses, the corresponding actual Mg and Ca doses were as follows: 17.5 mg/kg Mg and 12.1 mg/kg Ca in the L-HCM group; 35.1 mg/kg Mg and 24.2 mg/kg Ca in the M-HCM group; and 52.6 mg/kg Mg and 36.2 mg/kg Ca in the H-HCM group.

We employed both targeted and untargeted metabolomics experiments to analyze the intestinal metabolites of HCM. For untargeted metabolomics, thirty rats were divided into five groups: CON, TXA, L-HCM, M-HCM, and H-HCM. Twelve rats were assigned to two groups for targeted metabolomics: CON and H-HCM.

### 4.5. Identification of Key HCM Intermediate Metabolites by UHPLC-Q-Orbitrap HRMS Analysis

#### 4.5.1. Sample Pretreatments for the Detection of HCM Metabolites

For metabolomics analysis, forty-two rats were subjected to the same anesthesia and tail-amputation procedure for hemostasis assessment. At 2 h post-administration, the animals were euthanized by cardiac puncture followed by exsanguination. We took 0.5 mL samples of the small-intestinal contents, soaked them in 10 mL of 30% acetonitrile aqueous solution containing 1% formic acid, and subjected them to ultrasonication (KQ-250 DA, Kunshan Ultrasonic Instrument Co., Ltd., Kunshan, China) at 4 °C for 30 min. This extraction process was repeated twice to ensure thorough recovery of the HCM metabolites. The supernatants from both extractions were combined and centrifuged at 8944× *g* and 4 °C for 15 min for analysis.

Each sample was filtered through a 0.2 µm nylon membrane filter to identify HCM metabolites by UHPLC-Q-Orbitrap HRMS (Thermo Scientific, Waltham, MA, USA), equipped with a heated electrospray ionization (HESI) source in the positive and negative ionization modes following the procedure of previous studies with minor modifications [42].

#### 4.5.2. Untargeted Metabolomics Analysis of HCM Metabolites in Gastrointestinal Tracts

Compounds were separated on an ACQUITY UPLC ^®^ HSS T3 column (2.1 mm × 100 mm, 1.8 μm, Waters, Milford, MA, USA) at 30 °C. Then, 5 μL test samples were injected and separated by using a mobile phase consisting of acetonitrile (phase A) and 0.1% (*v*/*v*) formic acid (phase B) at a flow rate of 0.20 mL/min. The gradient elution scheme was as follows: 0–10 min, 2% A; 10–20 min, 2–20% A; 20–25 min, 20–50% A; 25–27 min, 50–90% A; and 27–30 min, 90–2% A. The column chamber and sample tray were maintained at 30 °C and 10 °C, respectively.

The optimal mass spectrometry (MS) parameters were a capillary temperature of 320 °C, a spray voltage of 3.5 kV in positive modes, and a spray voltage of 2.5 kV in negative modes. Nitrogen was used as the sheath and auxiliary gas at 30 and 10 psi flow rates, respectively. The auxiliary gas was then heated to 350 °C. The mass range was 70–1000 m/z, and the resolutions of the primary and secondary scans were 70,000 and 17,500, respectively. The normalized collision energy was 20 eV.

Untargeted metabolomics analysis of HCM metabolites in the small intestine was performed using the scanning mode, comprising Full MS and Full MS/dd–MS^2^. The mass spectrum data of the solvent used for sample preparation, the experimental group, and the TXA group, obtained from UHPLC-Q-Orbitrap HRMS, were imported into Compound Discoverer 3.2 software (Thermo Fisher Scientific, USA). The compounds in the solvent for sample preparation were deducted from the control group, the experimental group, and the TXA group. To ensure the accuracy of the data, we used two models ([H^+^] and [H^−^]) to separately search for the identities of the compounds in HCM metabolites via the built-in databases in Compound Discoverer 3.2 software (Thermo Fisher Scientific, Waltham, MA, USA), including mzCloud (https://www.mzcloud.org/, accessed on 21 December 2025) and ChemSpider (https://www.chemspider.com/, accessed on 21 December 2025), using formula or exact mass and secondary fragment ion information. Following peak extraction, alignment, denoising, and database searching, metabolic feature data were cleaned (missing-value handling, normalization, and standardization). PCA and OPLS-DA were then performed on the cleaned data to evaluate sample clustering and separation, followed by permutation testing to validate the OPLS-DA model. Differential metabolites were subsequently identified based on VIP values, statistical significance, and fold changes, and visualized using volcano plots and hierarchical clustering heatmaps.

#### 4.5.3. Targeted Metabolomics Analysis of HCM Metabolites in the Gastrointestinal Tracts

For the targeted metabolomics analysis of HCM in the small intestine, we used standards of twenty amino acids, TXA, and ACA. We subtracted the compounds in the solvent used for sample preparation for the control and the high-dose (H-HCM) groups. The analysis of the twenty amino acids, TXA, and ACA from HCM metabolites was performed using the scanning mode of Full MS and PRM. Chromatographic separation was carried out on an HILIC column (150 mm × 2.1 mm, 2.6 μm, Thermo Scientific, USA) at a temperature of 30 °C. We injected 5 μL of test samples into a mobile phase consisting of acetonitrile (phase A) and 0.005 mol/L ammonium acetate in water (phase B) at a flow rate of 0.20 mL/min. The gradient elution scheme was as follows: from 0 to 5 min, 100% to 70% A; from 5 to 15 min, 70% to 70% A; from 15 to 20 min, 75% to 50% A; from 20 to 23 min, 50% to 100% A; and from 23 to 25 min, 100% A.

The main optimal mass spectrum parameters, including molecular ions, major daughter ions, and ion monitoring mode, for twenty amino acids, TXA, and ACA, are summarized in Supplement Table S1. We used the peak areas of the compounds listed in Table S1 to create a heatmap for the control and high-dose (H-HCM) groups. For targeted metabolomics statistics, we present the following: (1) a heatmap generated from hierarchical clustering analysis, incorporating both positive and negative ion scanning modes, and (2) a summary of enrichment and pathway analyses conducted with MetaboAnalyst 5.0, comparing the control group to the high-dose group (H-HCM).

### 4.6. Hemostasis In Vivo

#### 4.6.1. Coagulation Index Analysis

For hemostasis and coagulation evaluation, seventy-two rats were deeply anesthetized via intraperitoneal injection of tribromoethanol (2.5%, 8 mL/kg). Under deep anesthesia, the tail was amputated 3 cm from the tip, and hemostasis time and blood loss were measured as described below. Blood samples were then collected via cardiac puncture, followed by exsanguination to ensure euthanasia. Rat plasma was prepared by combining 1.8 mL of whole blood with 0.2 mL of anticoagulant (sodium citrate, 183.5 mM) and then centrifuging the resulting solution for 10 min at 3000 g and 25 °C. The APTT, PT, TT, and FIB levels in the rat plasma were then quantified using an automated blood coagulation analyzer and a kit designed for this purpose [43]. All experiments were completed within 3 h after blood collection.

#### 4.6.2. Determination of Hemorrhage Volume and Hemostasis Time

Hemostasis time was determined by severing the rat’s tail 3 cm from the tip and monitoring the outflow of blood. Gauze was used to absorb blood every 5 s until clot formation occurred. The blood loss was quantified by weighing the gauze before and after blood absorption and calculating the difference in weight.

### 4.7. Statistical Analysis

All experimental data are expressed as mean ± SD. All assays were performed in sextuplicate. For untargeted metabolomics, we conducted multivariate analysis using principal component analysis (PCA) to visualize clustering patterns and identify outliers. We used orthogonal partial least squares-discriminant analysis (OPLS-DA) to identify discriminatory metabolites. The models were validated using permutation tests and cross-validation methods. All data were analyzed using one-way analysis of variance (ANOVA) with SPSS 20.0. A *p*-value of * *p* < 0.05 was considered significant, and ** *p* < 0.01 was considered very significant. We used Origin 2022 software to create the hierarchical clustering analysis heatmap and the bar chart for coagulation index analysis. Enrichment analysis and pathway analysis were summarized using MetaboAnalyst 5.0.

## 5. Conclusions

In this study, high-purity cuttlefish melanin was successfully isolated from cuttlefish ink sac by-products, demonstrating the potential of this underutilized marine resource for value-added utilization. SEM-EDS analysis showed that HCM consisted of black spherical nanoparticles enriched in calcium and magnesium. Multi-technique characterization (UV–Vis, FTIR, XPS, and Raman) confirmed the material as melanin, with characteristic optical absorption, surface functional groups, and elemental composition. In vivo evaluation indicated that HCM significantly reduced blood-loss volume and shortened bleeding time while maintaining acceptable biosafety, suggesting promising hemostatic activity. Mechanistically, metabolomics analysis detected TXA and ACA in the small intestine following HCM administration. Additionally, the release of Ca and Mg from HCM may contribute to regulating coagulation. Our findings suggest that HCM may exert its hemostatic effect through two systems: the plasmin/plasminogen (fibrinolysis) system and the coagulation system. However, this dual mechanism remains to be definitively established. Overall, this work provides mechanistic evidence for the hemostatic activity of cuttlefish melanin and supports the high-value utilization of cuttlefish ink sac by-products as a source of marine-derived functional ingredients.

## Data Availability

The data presented in this study are available on request from the corresponding authors.

## Supplementary Materials

The following supporting information can be downloaded at: https://www.mdpi.com/article/10.3390/molecules31203654/s1. Table S1: Identification of significant differential metabolites in the rat small intestine using UHPLC-Q-Orbitrap HRMS analysis; Table S2: Targeted metabolomic analysis of intestinal amino acids in CON and H-HCM rats. Figure S1: Hierarchical clustering heatmap of differential metabolites across the five groups in negative ion mode. CON: control group; TXA: positive administration group; L-HCM: Low-dose group; M-HCM: Medium-dose group; H-HCM: High-dose group.

## Abbreviations

The following abbreviations are used in this manuscript:

CM	Cuttlefish melanin
HCM	High-purity cuttlefish melanin
TXA	Tranexamic acid
ACA	6-aminocaproic acid
DHICA	5, 6-dihydroxyindole-2-carboxylic acid
DHI	5, 6-dihydroxyindole
UHPLC-Q-Orbitrap HRMS	Ultrahigh-performance liquid chromatography quadrupole-Orbitrap high-resolution mass spectrometry
SEM	Scanning electron microscopy
EDS	Energy-dispersive X-ray spectroscopy
ICP-OES	Inductively coupled plasma optical emission spectrometry
FTIR	Fourier transform infrared spectrometer
UV–Vis	Ultraviolet–visible
XPS	X-ray photoelectron spectroscopy
CON	Control group
HESI	Heated electrospray ionization
MS	Mass spectrometry
PCA	Principal Component Analysis
OPLS-DA	Orthogonal partial least squares-discriminant analysis
Bio-MOFs	Biological Metal–Organic Frameworks
APTT	Activated partial thromboplastin time
PT	Prothrombin time
TT	Thrombin time
FIB	Fibrinogen
