# Supplementary material for: Hemostatic Activity of Orally Administered Melanin from Cuttlefish Ink Sacs in a Rat Tail-Bleeding Model
Source: Molecules. 2026 Oct 13;31(20):3654. doi: 10.3390/molecules31203654 (PMC13707964; doi:10.3390/molecules31203654)

Table S1 Identification of significantly differential metabolites in the rat small intestine using UHPLC-Q-Orbitrap HRMS analysis.

No.	Compound ID	Metabolite identification	Formula	t_R (min)	Selected ion	Exact mass (Da)	Error(ppm)	Fragment ions	Change	Related pathway
									H-HCM(CON)	
1	HMDB0014447	TXA	C8H15NO2	9.98	[M+H]+	158.11756	0.189732	95.08603; 141.15106; 123.08055	↑**	
					[M-H]-	156.10300	0.768723	57.97446;85.00301; 89.02306		
2	HMDB0001901	ACA	C6H13NO2	9.83	[M+H]+	132.10191	0.151398	114.09141;69.07043; 96.08105	↑*	
					[M-H]-	130.08735	1.691171	61.98692;102.98723; 111.04371		
3	HMDB0000883	L-Val	C5H11NO2	6.69	[M+H]+	118.08626	2.20178	72.08142; 57.05801; 55.05497	↑	Val, Leu, and Ile biosynthesis
					[M-H]-	116.07170	2.756917	71.01250; 99.92423; 89.01333		
4	HMDB0000167	L-Thr	C4H9NO3	7.17	[M+H]+	120.06552	0.166576	74.06068;102.05531; 56.05024	↑*	Val, Leu, and Ile biosynthesis
					[M-H]-	118.05097	0.762383	74.02335; 72.00770; 109.54774		
5	HMDB0000159	L-Phe	C9H11NO2	5.95	[M+H]+	166.08626	0.240839	120.08094； 103.05449； 131.04910	↑	Phe, Tyr, and Try biosynthesis
					[M-H]-	164.07170	1.036132	119.04889； 147.04382； 72.00771		
6	HMDB0000517	L-Arg	C6H14N4O2	9.56	[M+H]+	175.11895	4.968052	158.08116; 129.10231; 84.04491	↑*	Arg biosynthesis

					[M-H]-	173.10440	4.448183	155.08128; 128.03397; 84.04409		
7	HMDB0000182	L-Lys	C6H14N2O2	9.74	[M+H]+	147.11280	0.475825	84.04494；102.05531；130.04993	↓	Biotin metabolism
					[M-H]-	145.09825	0.089594	102.05463； 128.03397；129.47148		
8	HMDB0000158	L-Tyr	C9H11NO3	6.28	[M+H]+	182.08117	0.384444	136.07568; 165.05458; 123.04420	↑	Phe, Tyr, and Try biosynthesis
					[M-H]-	180.06662	4.66494	163.03879; 119.04886; 93.03321		
9	HMDB0000161	L-Ala	C3H7NO2	6.83	[M+H]+	90.05495	0.222087	62.17123;72.04501; 87.00449	↑**	Ala, Asp, and Glu metabolism
					[M-H]-	88.04040	2.498853	60.99170;71.01242; 59.01243		
10	HMDB0000187	L-Ser	C3H7NO3	7.73	[M+H]+	106.04987	0.377181	60.04514; 88.03979; 70.02937	↓	Glyoxylate and dicarboxylate metabolism
					[M-H]-	104.03532	0.961212	87.92390;72.00771; 74.02335		
11	HMDB0000929	L-Try	C11H12N2O2	6.94	[M+H]+	205.09715	0.877633	120.08084; 166.08607; 188.07047	↓	Try metabolism
					[M-H]-	203.08260	5.071828	164.07048; 132.02892;61.98700		
12	HMDB0000172	L-Ile	C6H13NO2	6.13	[M+H]+	132.10191	0.075699	86.09694; 69.07048; 61.01134	↓	Val, Leu, and Ile biosynthesis
					[M-H]-	130.08735	4.535414	95.02364; 114.01823; 70.02843		
13	HMDB0000191	L-Asp	C4H7NO4	8.21	[M+H]+	134.04478	4.699922	87.02685; 133.03189; 74.02430	↑	Arg biosynthesis; His metabolism
					[M-H]-	132.03023	4.620154	88.03896; 115.00226; 71.01247		

14	HMDB0060475	L-Glu	$C_5H_9NO_4$	8.09	[M+H]+	148.06043	1.21572	84.04491; 102.05453; 130.04990	↓	Arg biosynthesis; His metabolism
					[M-H]-	146.04588	4.519128	102.05469; 128.03400; 61.98703		
15	HMDB0000177	L-His	$C_6H_9N_3O_2$	9.23	[M+H]+	156.07675	0.256284	110.07158; 95.06078; 83.06092	↓	His metabolism
					[M-H]-	154.06220	4.803255	137.03429; 93.04441; 72.00768		

↑: Upregulated; ↓: Downregulated; *: Statistically significant (P < 0.05); **: Highly statistically significant (P < 0.01); tR: retention time; Con: control group; TXA: positive administration group; H-HCM: High-dose group; TXA: Tranexamic acid; ACA: 6-Aminocaproic acid; Val: Valine; Thr: Threonine; Phe: Phenylalanine; Arg: Arginine; Lys: Lysine; Tyr: Tyrosine; Ala: Alanine; Ser: Serine; Try: Tryptophan; Ile: Isoleucine; Asp: Aspartic acid; Glu: Glutamate; His: Histidine.

Table S2 Targeted metabolomic analysis of intestinal amino acids in CON and H-HCM rats.

No.	Metabolite identification	Formula	CON (μM)	H-HCM (μM)
1	TXA	C8H15NO2	/	769.12±100.79
2	ACA	C6H13NO2	/	2756.64±309.25
3	L-Val	C5H11NO2	18658.53±1932.43	33209.56±9539.71
4	L-Thr	C4H9NO3	1572.15±119.52	2685.70±599.02
5	L-Phe	C6H13NO2	223.95±68.94	343.67±127.98
6	L-Arg	C4H7NO4	1742.11±49.59	2618.90±199.78
7	L-Lys	C6H14N2O2	3141.87±966.50	3052.45±725.16
8	L-Tyr	C5H9NO4	1008.42±134.58	1477.32±260.52
9	L-Ala	C6H9N3O2	2570.25±688.07	1815.47±1710.94
10	L-Ser	C9H11NO2	681.64±74.12	518.28±140.31
11	L-Try	C6H14N4O2	794.45±74.03	379.75±134.58
12	L-Ile	C9H11NO3	6065.12±436.82	5082.81±798.56
13	L-Asp	C3H7NO2	1255.45±123.41	1189.92±80.05
14	L-Glu	C3H7NO3	5513.54±226.97	5020.96±532.11
15	L-His	C11H12N2O2	1622.63±355.01	986.32±253.74

Figure S1. Hierarchical clustering heatmap of differential metabolites across the five groups in negative ion mode. CON: control group; TXA: positive administration group; L-HCM: Low-dose group; M-HCM: Medium-dose group; H-HCM: High-dose group.

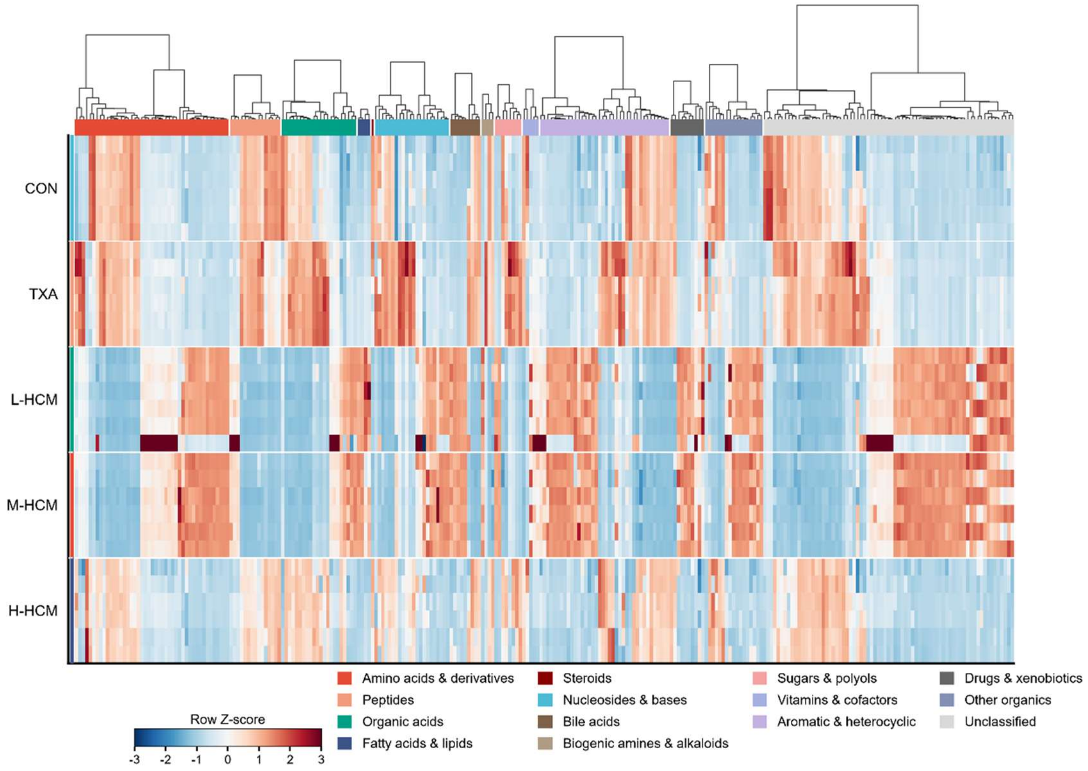

Supplement: Supplementary file 1 [file molecules-31-03654-s001.zip › molecules-4469497-supplementary.pdf]
